# The Abiotic Formation of Pyrrole under Volcanic, Hydrothermal Conditions—An Initial Step towards Life’s First Breath?

**DOI:** 10.3390/life11090980

**Published:** 2021-09-17

**Authors:** Christian Seitz, Wolfgang Eisenreich, Claudia Huber

**Affiliations:** Bayerisches NMR Zentrum, Strukturelle Membranbiochemie, Department Chemie, Technische Universität München, Lichtenbergstr. 4, 85748 Garching, Germany; c.seitz@tum.de (C.S.); wolfgang.eisenreich@mytum.de (W.E.)

**Keywords:** pyrrole, dimethylpyrrole, acetylene, propyne, transition metal sulfides, hydrothermal conditions, origin of life

## Abstract

Porphyrins, corrins, and tetrapyrroles constitute macrocycles in essential biomolecules such as heme, chlorophyll, cobalamin, and cofactor F430. The chemical synthesis as well as the enzymatic synthesis of these macrocycles starts from pyrrole derivatives. We here show that pyrrole and dimethyl pyrrole can be formed under the simulated volcanic, hydrothermal conditions of Early Earth, starting from acetylene, propyne, and ammonium salts in the presence of NiS or CoS as catalysts.

## 1. Introduction

Tetrapyrroles can be found in almost all modern domains of life with important functions, being mainly involved in respiration (heme), photosynthesis (chlorophyll), amino acid synthesis (cobalamin), and methane formation (cofactor F430) [1,2,3].

**Figure 1:**
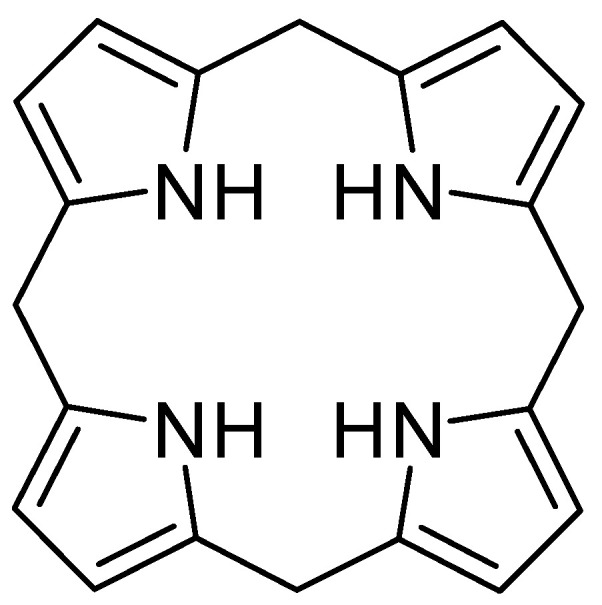
Structure of porphyrinogen, the most simple, non-aromatic tetrapyrrole.

The basic structure of tetrapyrroles can be seen in Figure 1. The structures of heme, chlorophyll, cobalamin, and cofactor F430 are shown in the supplemental section (Figures S1–S4).

The chemical synthesis of pyrrole and its derivatives was one of the early milestones in classical organic chemistry. *Ludwig Knorr* discovered in the 1880s that α-amino ketones (1) and a compound with an electron-withdrawing group next to a carbonyl group (2) yield the corresponding substituted pyrroles (*Knorr* pyrroles) (3) [4]. In the 1940s, *Hans Fischer* and *Emmy Fink* discovered that the *Knorr* pyrroles (3) are not the only products of this reaction. With a second carbonyl group (e.g., β-ketoesters) in the non-amino educt, a mixture of the Knorr (3) and the *Fischer–Fink* products (4) could be observed (Schemes S1–S3) [5]. *Kleinspehn* further designed the reaction parameters so that the *Fischer–Fink* product is the exclusive reaction product [6].

The *Paal–Knorr* reaction, developed in 1884, is another example of the formation of a pyrrole derivative [7,8]. Here, a 1,4-diketone (5) reacts with an amine (6) to produce the corresponding pyrrole derivate (7) (Schemes S4 and S5). Alternatively, α-haloketones (8) can be reacted with ammonia (or primary amines) (6) and β-ketoesters (9) to afford the corresponding pyrroles (10) (Hantzsch pyrrole synthesis, Schemes S6 and S7) [9].

**Scheme 1:**
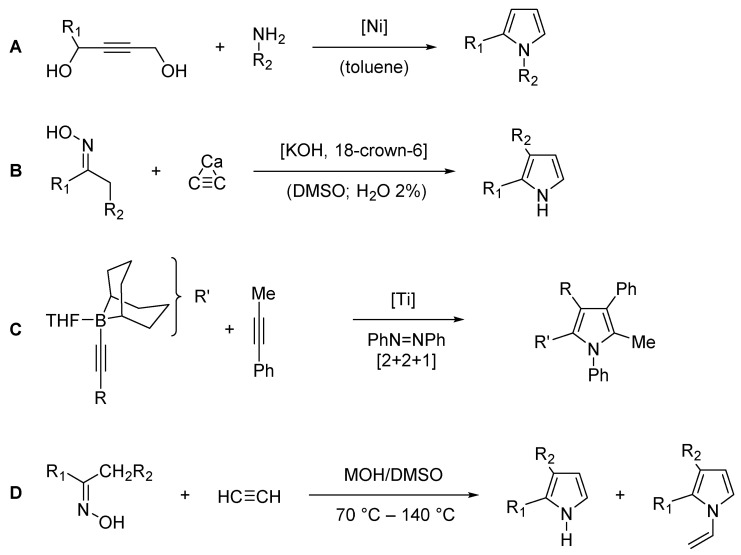
Examples of modern-day pyrrole synthesis. (**A**) Pyrrole synthesis with nickel as catalyst [11]; (**B**) pyrrole synthesis with calcium carbide [12]; (**C**) pyrrole synthesis with two acetylene-like compounds [13]; (**D**) pyrrole synthesis with acetylene [10].

Next to these classical reactions, pyrroles can also be synthesized from acetylene or acetylene-like molecules as one of the starting materials [10]. In the following, four examples are shown which are, in some aspects, related to our setting. More specifically, nickel was used as a catalyst for the condensation of an acetylene derivative with an amine (Scheme 1A) [11]. The reaction can also be performed with acetylene generated in situ from calcium carbide (Scheme 1B) [12], or by using one nitrogen compound and two acetylene-like compounds (Scheme 1C) [13]. In the setting used by *Trovimov* et al. (Scheme 1D), the pyrrole ring was built from ketones (ketoximes) and acetylene in superbasic catalytic systems, finally leading to a variety of pyrrole-like structures through variations in the ketoxime side chains [10].

All examples use very specific catalysts and non-aqueous solvents to form the corresponding pyrrole derivatives. In contrast with these conditions, our reactions are performed from simple, prebiotic, relevant building blocks in an aqueous medium.

Acetylene can be either formed in volcanic processes [14], or by the reaction of CaC_2_ with water [15]. Acetylene has also been detected in extant fumarolic gases [16] and in volcanic glasses [17]. Atmospheric acetylene has been suggested as part of a presumptive methane-rich primordial atmosphere due to photolysis of methane [18]. Acetylene is highly reactive and shows strong ligation to transition metals. It is therefore considered as a possible carbon source for the formation of biomolecules in prebiotic scenarios. Propyne (methylacetylene) can be found besides acetylene in volcanic gas samples [14], can be formed from propylene through pyrolysis [19], and is also detected in interstellar cold clouds [20].

In previous work, we showed the formation of short-chain fatty acids [21] and intermediates of extant carbon fixation cycles [22] from acetylene and carbon monoxide as carbon sources under simulated volcanic hydrothermal conditions.

By adding ammonium chloride as a nitrogen source, we now show that pyrrole is formed out of acetylene and ammonia in the presence of NiS and CoS at 105 °C after 1 day. Transition metal sulfides served as catalysts, according to *Günter Wächtershäuser’s* Iron–Sulfur World theory [23]. This proof-of-principle reaction could provide the basis for the prebiotic synthesis of more complex pyrroles, including tetrapyrroles or related molecules.

## 2. Materials and Methods

All chemicals were purchased from Sigma-Aldrich GmbH (Steinheim, Germany) in the highest purity available. Acetylene 2.6 (acetone free) was purchased from Linde AG (Pullach, Germany), and CO 2.5 and argon 4.6 were purchased from Westfalen AG (Münster, Germany). In a typical run (run 1, Table S1), a 125 mL glass serum bottle was charged with 1.0 mmol NiSO_4_·6 H_2_O and 1.0 mmol NH_4_Cl, and closed with a gas-tight silicon stopper. The bottle was evacuated three times and filled with argon, finally resulting in a de-aerated state. Subsequently, the bottle was filled with 1 M Na_2_S solution, 1 M NaOH solution, and argon-saturated water, resulting in a total reaction volume of 5 mL. In this mixture, a precipitate of black NiS was immediately formed due to its low solubility constant of 1 × 10^−22^ [15,24,25] in aqueous solution. Finally, 60 mL of acetylene gas and 60 mL of CO were added. Liquids and gases were injected by using gas-tight syringes. The freshly precipitated NiS (NiSO_4_ + Na_2_S → NiS↓ + Na_2_SO_4_) acted as a putative transition metal catalyst for the reaction, and the molar variations of Na_2_S to NiSO_4_ resulted in free sulfide ions in the solution. For the formation of dimethylpyrrole, propyne was used instead of acetylene.

Reactions were carried out at 105 °C. pH variations were achieved through the addition of H_2_SO_4_ solution (1 M), NaOH solution (1 M), and solid Ca(OH)_2_. For safety reasons (danger of explosion) and for technical reasons, the reactions were carried out at low gas pressure. Bottles were filled with ~1 bar at room temperature, leading to a total absolute pressure of ~2.5 bar at 105 °C. After the defined reaction time, the reaction mixture was allowed to cool down and transferred into a test tube. After centrifuging at 3000 rpm for 10 min, the supernatant was extracted with 3 × 1 mL ethyl acetate. The organic phases were dried over Na_2_SO_4_ and directly analyzed with gas chromatography–mass spectrometry (GC–MS). GC–MS analysis was performed with a GC-2010, coupled with MS-QP2010 Ultra (Shimadzu GmbH, Duisburg, Germany) with a 30 m × 0.25 mm × 0.25 µm fused silica capillary column (Equity TM5, Supelco, Bellefonte, PA, USA) and an AOC-20i auto injector. Additionally, 1 mL of supernatant was freeze dried and treated with 250 µL ACN and N-(tert-butyldimethylsilyl)-N-methyltrifluoracetamide each, and heated at 70 °C for about an hour.

Temperature program and settings for ethyl acetate phase: 0–6 min at 40 °C; 6–25 min at 40–280 °C, 10 °C/min; injector temperature, 260 °C; interface temperature, 260 °C; column flow rate, 1 mL/min; scan interval, 0.5 s; and injection volume, 1.0 µL.

Temperature program and settings for silylated products: 0–6 min at 90 °C; 6–25 min at 90–310 °C, 10 °C/min; injector temperature, 260 °C; interface temperature, 260 °C; column flow rate, 1 mL/min; scan interval, 0.5 s; and injection volume, 0.5 µL.

Peak assignment was achieved by comparison with the retention times and mass spectra of purchased reference compounds, as well as with data from the National Institute of Standards and Technology (NIST) spectral library. Pyrrole showed a retention time of 5.4 min; dimethylpyrrole a retention time of 8.8 min. Quantification was performed by external calibration using known concentrations of pyrrole and dimethylpyrrole, respectively. Runs without a transition metal compound or with argon instead of acetylene were performed for comparison.

## 3. Results

**Scheme 2:**
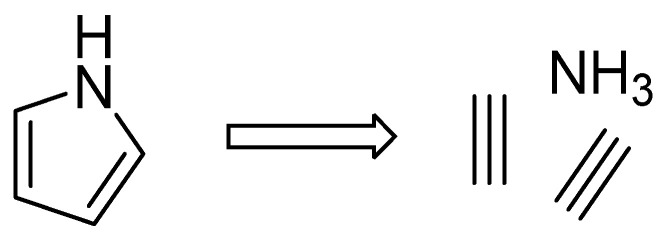
Retrosynthesis of pyrrole, formed by two molecules of acetylene (≡) and NH_3_.

We here show the formation of pyrrole under simulated volcanic hydrothermal conditions from simple and geochemically feasible building blocks. In a retrosynthetic view, two molecules of acetylene and one molecule of NH_3_ forms pyrrole (Scheme 2).

**Figure 2:**
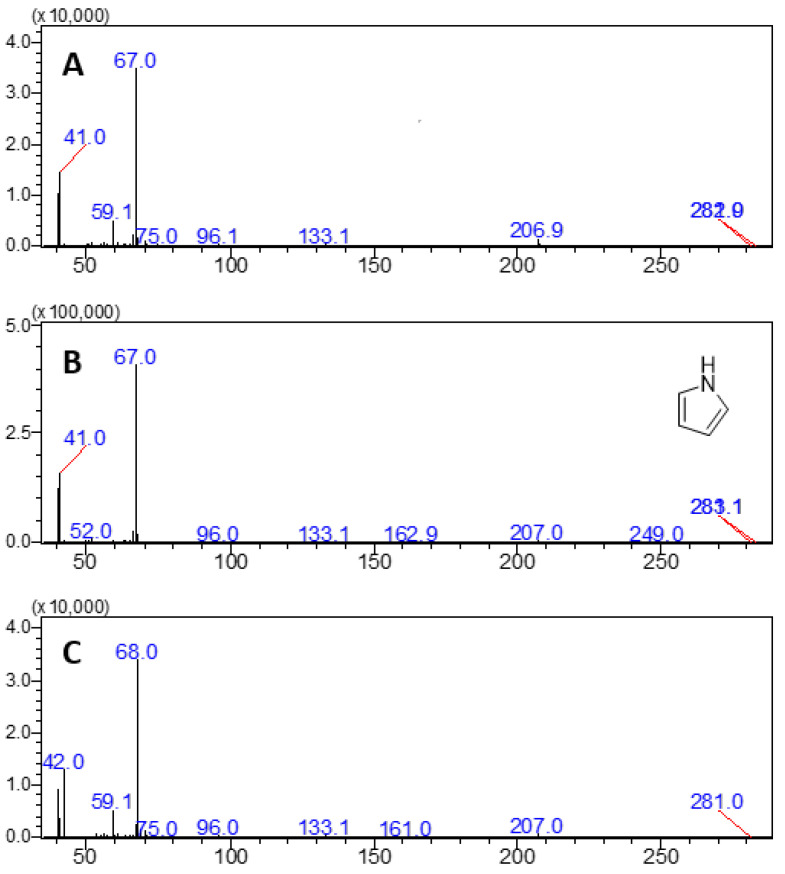
Mass spectra of pyrrole with 67.0 u as the molecule mass and 41.0 u as the mass of the characteristic fragment C-C=N^+^. (**A**) Pyrrole spectrum from run 1 (Table 1). (**B**) Pyrrole spectrum from standard. (**C**) Pyrrole spectrum from a run performed with ^15^NH_4_Cl.

Using our GC–MS settings, pyrrole was found at a retention time of 5.6 min (65.9 °C), as shown by comparison with the reference material. The highest mass peak was observed at 67.0 u. A second characteristic peak was detected at 41.0 u, which belongs to the C-C=N^+^ fragment. When the stable isotope labeled ^15^NH_4_Cl was used instead of unlabeled ^14^NH_4_Cl, the detected product displayed a molecule mass +1 in the corresponding GC–MS analysis (Figure 2).

NiS was used as a transition metal catalyst for the formation of pyrrole from acetylene and ammonia. Run 1 (composition: 1 mmol NiSO_4_, 1 mmol Na_2_S, 1 mmol NH_4_Cl, 1 mL 1 M NaOH, 60 mL C_2_H_2_, 60 mL CO, 105 °C) (see Table 1) was set as the standard run with a maximum yield of 1.14% pyrrole, based on the conversion of NH_4_Cl. In runs without a transition metal, acetylene, or nitrogen source, but otherwise identical conditions to run 1, no pyrrole formation was observed.

FeS and CoS were tested as further transition metal catalysts for pyrrole formation. In these cases, FeSO_4_ and CoSO_4_ were used instead of NiSO_4_. The pyrrole formation in experiments using cobalt was about 50% compared to experiments with nickel. In the case of iron, no formation of pyrrole could be observed (Table 1).

In the above-described setup, acetylene was present with a small molar excess compared to NH_4_Cl (2.57 mmol to 1.00 mmol). When the amount of acetylene was increased to 120 mL (5.14 mmol), the yield of pyrrole decreased to 50% as compared to run 1, indicating that pyrrole formation was not limited by C_2_H_2_ in our setting.

**Figure 3:**
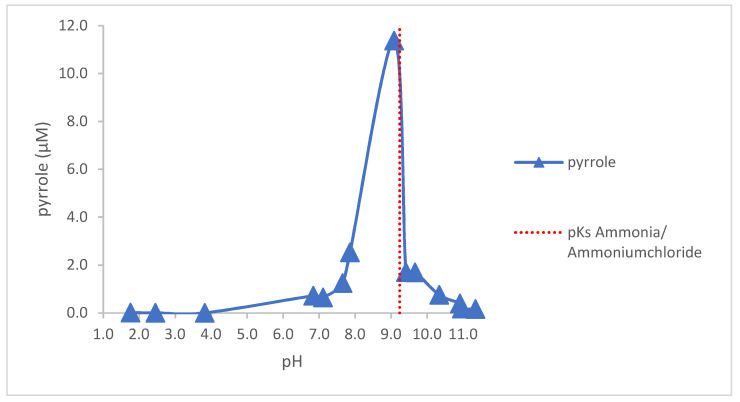
Formation of pyrrole at different pH values. pH values were measured at the end of the reaction time. The red dotted line shows the pKa of NH_3_/NH_4_Cl.

The yield of pyrrole had its optimum around pH 9.1 (Figure 3; Table S1), which corresponds to the pKa of ammonia of 9.25. At acidic pH values, pyrrole was not detected. At higher pH values than the pKa of ammonia, the yields decreased rapidly (Figure 3). This observation could be explained by higher concentrations of OH^−^ and therefore a blockage of catalytic nickel sites. This is supported by runs, with Ni(OH)_2_ instead of NiSO_4_, under otherwise identical parameters, which show no formation of pyrrole (Table 1).

**Figure 4:**
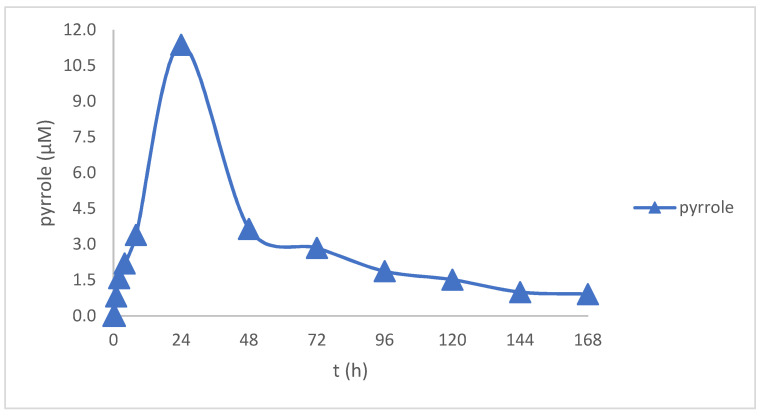
Time-dependent NiS-catalyzed formation of pyrrole from acetylene and ammonia.

Pyrrole formation was observed in reaction times from 0 min to 7 days (168 h). Notably, pyrrole formation could already be observed after the short reaction time of one hour, and showed its maximum after one day (24 h) (Figure 4; Table S2). A longer reaction period led to continuously declining yields of pyrrole. This observation indicates follow-up reactions of pyrrole, which, under optimized conditions, combined with suitable reactants, could lead to the required oligomerization of pyrrole to biologically important cyclic tetrapyrroles (see Figures S1–S4).

**Figure 5:**
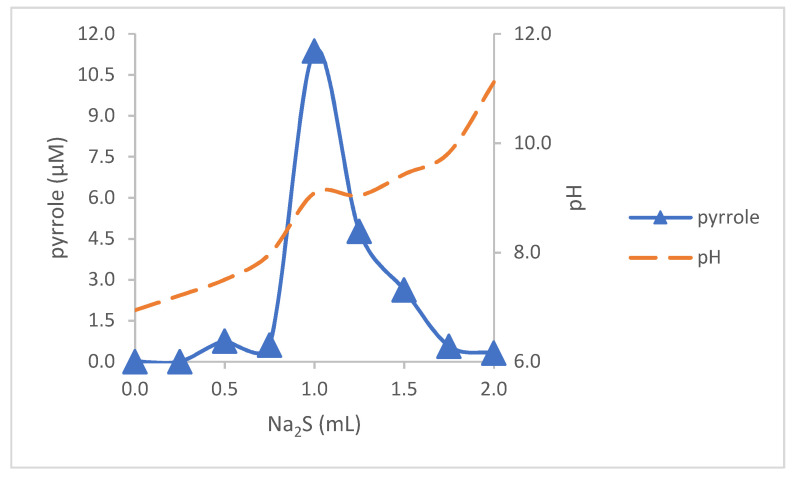
Formation of pyrrole with different concentrations of Na_2_S. The orange line indicates the pH of the corresponding experiment.

The concentration of catalysts also played an important role. Out of the possible nickel compounds in our experimental setup, NiSO_4_, NiOH, and NiS, only NiS supported pyrrole formation (Table 1, Table S3). Therefore, we changed the amount of Na_2_S in some runs to achieve different concentrations of NiS. The maximum yield of pyrrole was found at an equimolar concentration to the transition metal (Figure 5, Table S3). As mentioned earlier, a high pH inhibited the formation of pyrrole. Therefore, high concentrations of Na_2_S hindered the formation of pyrrole by increasing the pH of the solution. On the other hand, with NiSO_4_ not catalytically active, a low concentration of Na_2_S showed lower yields of pyrrole, since NiS could not be formed.

**Scheme 3:**
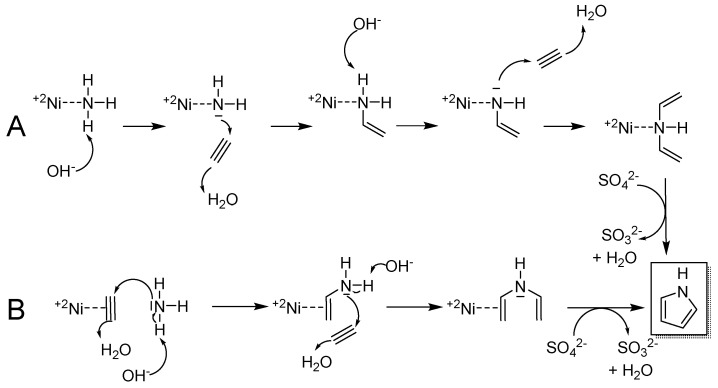
Proposed mechanism of the Ni-catalyzed formation of pyrrole. (**A**) starts with the binding of ammonia to the Ni-ion, (**B**) starts with an acetylene–nickel bond.

The classical pyrrole syntheses mentioned above involve the nucleophilic attack of nitrogen at a carbonyl group, resulting in the elimination of water. In our experimental setting, this mechanism would also be possible via the formation of carbonyl groups from CO. However, the formation of pyrrole was also observed in the absence of CO. Due to this result, two mechanisms for the formation of pyrrole can be suggested. The first one would be similar to the possible mechanism for the formation of thiophene, which was published earlier [26,27]. Here, NH_3_ displaces the sulfide ion from the transition metal and therefore acts as the reactant (Scheme 3A). After two steps of deprotonation and binding to two acetylene molecules, the corresponding divinyl amine undergoes an oxidative ring closure. At this stage, SO_4_^2−^ is possibly reduced to SO_3_^2−^, which can be found in the freeze dried liquid after silylation (Figure S5). Another possibility of an oxidizing agent in our oxygen-free reaction mixture would be the reduction of Ni^2+^ to Ni^0^. The formation of Ni^0^ from Ni^2+^ was shown earlier in similar experiments [28].

Another possible mechanism starts with acetylene binding to the Ni ion (Scheme 3B). Notably, acetylene shows strong coordination tendency towards Ni ions [29,30]. The following reaction steps would be identical; namely, two deprotonations, binding to two molecules of acetylene, and ring closure.

**Figure 6:**
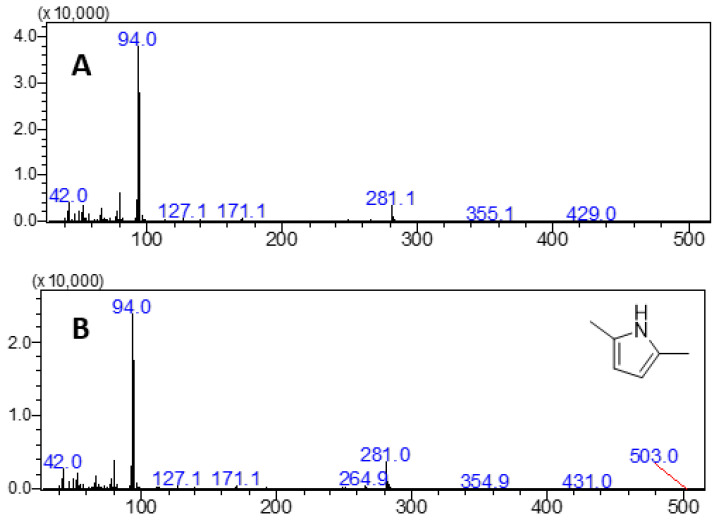
Mass spectra of dimethylpyrrole with 94.0 u as the molecule, and 80.0 u as the mass resulting of the loss of a methyl group. (**A**) First spectrum is of run 7 (Table 1). (**B**) Dimethylpyrrole spectrum from standard.

The oligomerization of pyrrole to porphin rings would require an additional CH group. As a proof of principle towards the formation of these derivatives, we tested propyne (methylacetylene) instead of acetylene under experimental conditions, which led to the formation of pyrrole. Indeed, one of the products in this reaction was dimethylpyrrole. Dimethylpyrrole can be found at a retention time of 8.8 min. The typical masses were 95 u, which is the molecular peak, 94 u as M-1, and 80.0 u, which results from the loss of one methyl group (Figure 6). According to our mechanistic view, we assume that the position of the two methyl groups was at C2 and C5. However, products with other positions are possible. The dimethylpyrrole yield, based on the conversion of NH_4_Cl, was 13.2%, which is about ten times higher compared to the yield of pyrrole from acetylene.

In addition to pyrrole and dimethylpyrrole, further nitrogen-containing molecules were detected in trace amounts in single runs; for example, methylthiazole and pyridinol (Figures S6 and S7).

## 4. Discussion

We here showed that pyrrole and dimethyl pyrrole can be abiotically formed from acetylene or methylacetylene, ammonium chloride, and metal sulfides under aqueous conditions at 105 °C. The best yields for pyrrole were achieved at a pH of 9.1, an equimolar concentration of Na_2_S and NiSO_4_, and after one day of reaction time. The conditions of our experiments were chosen to fit a scenario where life possibly emerged near volcanic exhalations [31] or hydrothermal vents at hadean times [32]. Nowadays, for the synthesis of pyrrole derivatives, very specific educts, solvents, and catalysts are used, in an attempt to optimize yields. In contrast, the currently shown synthesis of pyrrole is based on simple starting materials under aqueous and oxygen-free reaction conditions, in order to simulate prebiotic environments. As mentioned above, iron, nickel, and cobalt are still part of important biomolecules in modern biochemistry. It is therefore tempting to speculate that they were also involved in the prebiotic synthesis of pyrrole and its polymers. In support of this hypothesis is the fact that iron, nickel, and cobalt are commonly found in the crust of the Earth [33,34]. In our experiments, NiS and CoS showed their potential for catalytic activity in the formation of pyrrole from acetylene and ammonia.

The most common belief regarding the atmosphere on early Earth is that it mostly consisted of CO_2_ and water vapor [35,36], with only small traces of N_2_ and CO. Ammonia, which does not fit in this highly oxidized atmosphere, could be formed out of NO_3_^−^ due to reduction driven by FeS/H_2_S [37]. Nitrate, in this scenario, could be formed from atmospheric N_2_ and CO_2_ by electric discharges under oxygen-free conditions [38] and subsequently be dissolved in the ancient ocean.

The yields of pyrrole and dimethylpyrrole in our experiments are comparatively low. For safety reasons, due to the danger of explosion, experiments were performed at low pressure. At sub-seafloor level (>1000 bar), yields would be increased [39,40].

The synthesis of pyrrole can be seen as a first step in the formation of larger porphyrin systems. A first achievement in this direction could represent the synthesis of dimethylpyrrole in our experiments when starting from propyne instead of acetylene. Different side chains could then be achieved by using the corresponding starting materials, either to enhance the reactivity of the molecule for polymerization, or to match the side chains of biological tetrapyrrole structures. As transition metal sulfides first served as catalysts in this reaction system, a later substitution of the sulfide through the newly formed pyrrole structures could be imagined for the evolution of the potent catalyst factor F430, cobalamin, and also at a later stage, when oxygen was available, hemoglobin and chlorophyll.

## Figures and Tables

**Table 1:** Pyrrole formation from acetylene and ammonia using different transition metals as catalysts and different gas compositions after one day at 105 °C. Metal sulfides were formed in situ from MeSO_4_ and Na_2_S. pH values were adjusted with 1 mL 1 M NaOH and measured at the end of the reaction time. Yields are based on the conversion of NH_4_Cl. Dimethylpyrrole was formed from propyne instead of acetylene.

Run	NiS	FeS	CoS	Ni(OH)_2_	NaOH	NH_4_Cl	CO	C_2_H_2_	Propyne	t_reac_	pH	Pyrrole (Runs 1–5) Dimethylpyrrole (Run 6)	Yield
	(mmol)	(mmol)	(mmol)	(mmol)	(mmol)	(mmol)	(mL)	(mL)	(mL)	(d)		(µM)	(%)
**1**	1	-	-	-	1	1	60	60	-	1	9.1	11.38	1.14
**2**	-	1	-	-	1	1	60	60	-	1	9.1	<0.1	<0.01
**3**	-	-	1	-	1	1	60	60	-	1	8.7	5.31	0.53
**4**	-	-	-	-	1	1	60	60	-	1	9.9	<0.1	<0.01
**5**	-	-	-	1	1	1	60	60	-	1	9.8	<0.1	<0.01
**6**	1	-	-	-	1	1	-	120	-	1	8.6	5.75	0.57
**7**	1	-	-	-	1	1	60		60	1	9.3	132.25	13.2

## Data Availability

Data are only reported in this publication including the Supplementary Materials file.

## Supplementary Materials

The following are available online at https://www.mdpi.com/article/10.3390/life11090980/s1, Figure S1: Structural formula of heme b, Figure S2: Structural formula of chlorophyll b, Figure S3: Structural formula of cobalamin (vitamin B12), Figure S4: Structural formula of coenzyme F430, Figure S5. Mass spectra of bis(tert-butyldimethylsilyl)-sulfite, Figure S6. Mass spectra of 2-methylthiazol, Figure S7. Mass spectra of 3-tert-butyldimethylsilyloxypyridine, Scheme S1: Knorr pyrrole synthesis, Scheme S2: Mechanism of Knorr pyrrole synthesis, Scheme S3: Mechanism of Fischer–Fink pyrrole synthesis, Scheme S6: Paal–Knorr reaction, Scheme S7: Mechanism of Paal–Knorr pyrrole synthesis, Scheme S4: Pyrrole synthesis according to Hantzsch, Scheme S5: Mechanism of Hantzsch pyrrole synthesis, Table S1: Formation of pyrrole depending on pH, Table S2: Formation of pyrrole depending on reaction time, Table S3: Formation of pyrrole depending on concentration of catalyst.
