# Supplementary material for: The Abiotic Formation of Pyrrole under Volcanic, Hydrothermal Conditions—An Initial Step towards Life’s First Breath?
Source: Life (Basel). 2021 Sep 17;11(9):980. doi: 10.3390/life11090980 (PMC8471139; doi:10.3390/life11090980)
Supplement: Supplementary file 1 [file life-11-00980-s001.zip › life-1386724-supplementary.pdf]

Supplement Materials

Figure S1. Structural formula of heme b 1
Figure S2. Structural formula of chlorophyll b. 2
Figure S3. Structural formula of cobalamin (vitamin B12). 2
Figure S4. Structural formula of coenzyme F430. 3
Figure S5. Mass-spectra of Bis(tert-butyldimethylsilyl)-sulfite 3
Figure S6. Mass-spectra of 2-methylthiazol 4
Figure S7. Mass-spectra of 3-tert-Butyldimethylsilyloxypyridine. 4

Scheme S1. *Knorr*-pyrrole synthesis 5
Scheme S2. Mechanism of the *Knorr*-pyrrole-synthesis 5
Scheme S3. Mechanism of the *Fischer-Fink*-pyrrole-synthesis 6
Scheme S4. *Paal-Knorr*-reaction. 6
Scheme S5. Mechanism of the *Paal-Knorr*-pyrrole-synthesis 6
Scheme S6. Pyrrole synthesis according to *Hantzsch*. 7
Scheme S7. Mechanism of the *Hantzsch'sche*-pyrrole-synthesis 7

Table S1. Formation of pyrrole depending on pH. 8
Table S2. Formation of pyrrole depending on reaction time 8
Table S3. Formation of pyrrole depending on concentration of catalyst. 9

Figures

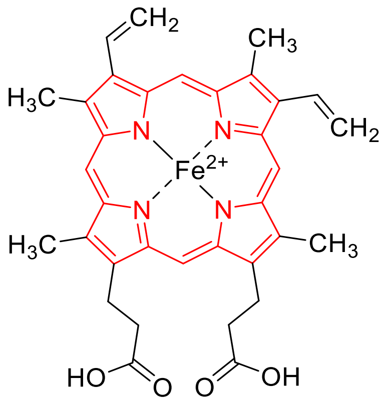

Figure S1. Structural formula of heme b. Tetrapyrrole is indicated in red

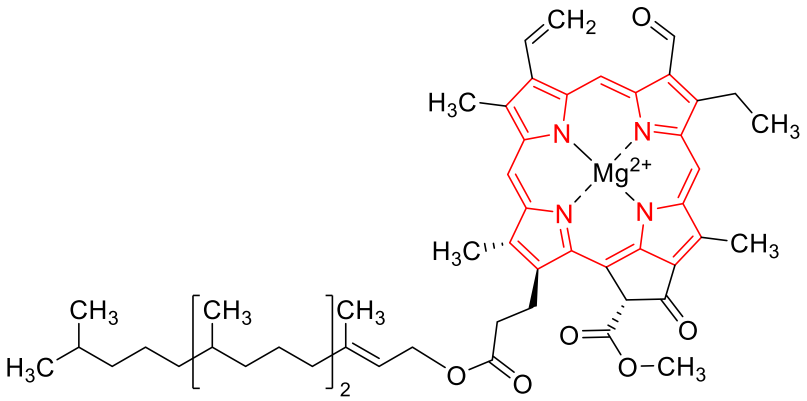

Figure S2. Structural formula of chlorophyll b. Tetrapyrrole is indicated in red

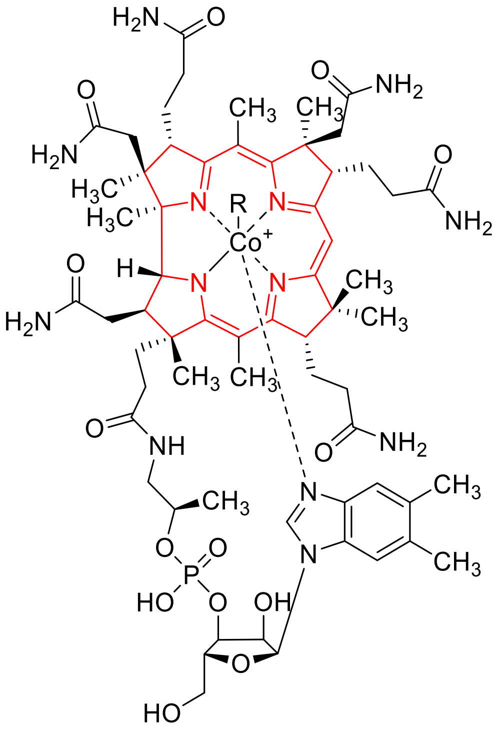

Figure S3. Structural formula of cobalamin (vitamin B12). Tetrapyrrole is indicated in red

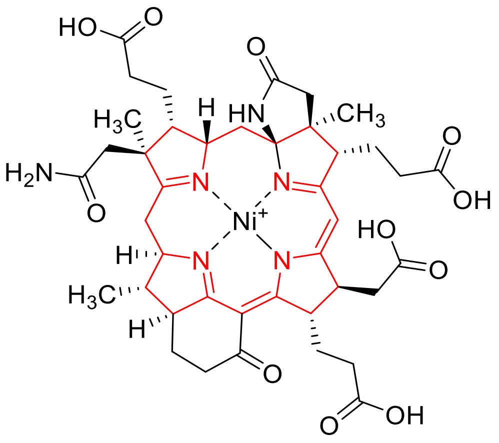

Figure S4. Structural formula of coenzyme F430. Tetrapyrrole is indicated in red

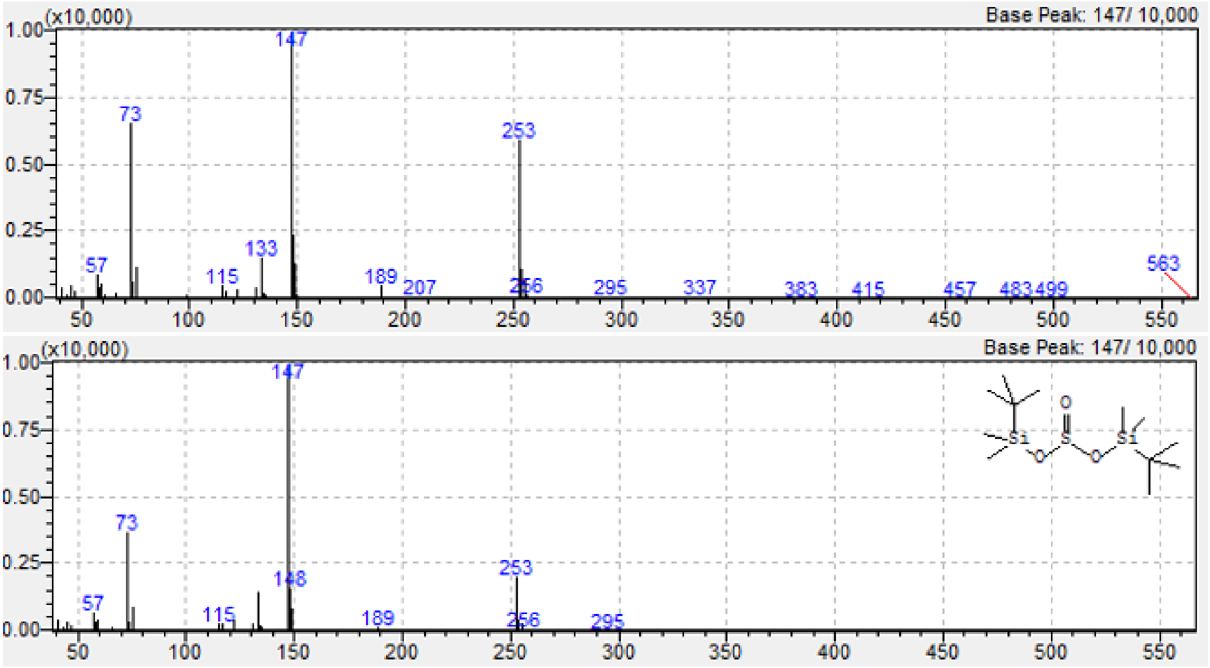

Figure S5. Mass-spectra of Bis(tert-butyldimethylsilyl)-sulfite. First spectrum is of a typical run, second spectrum is from NIST.

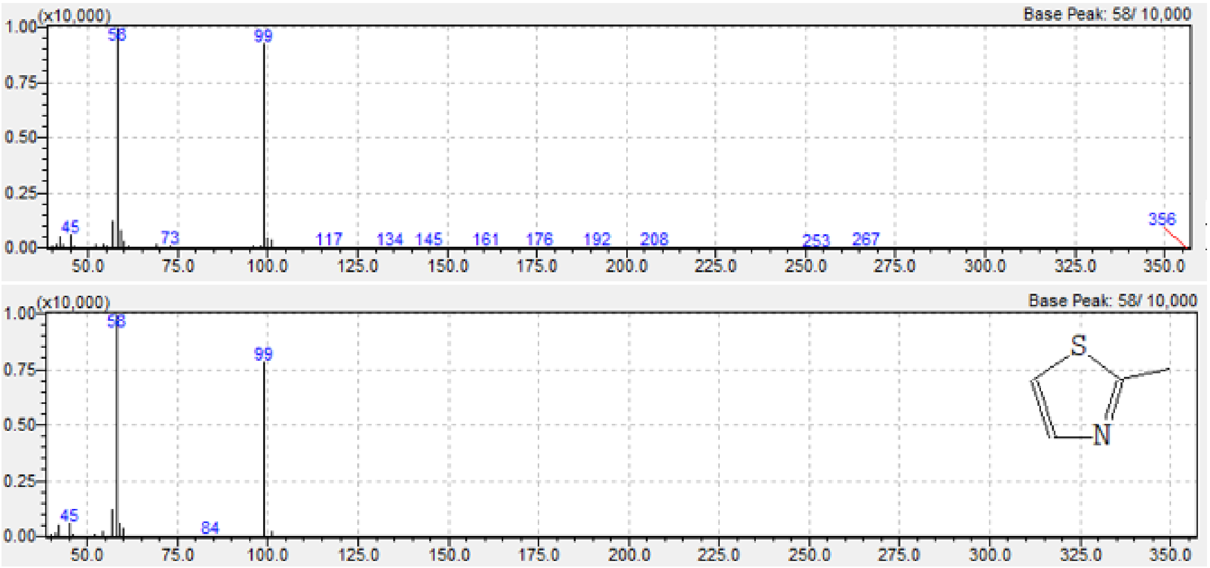

Figure S6. Mass-spectra of 2-methylthiazol. First spectrum is of a typical run, second spectrum is from NIST.

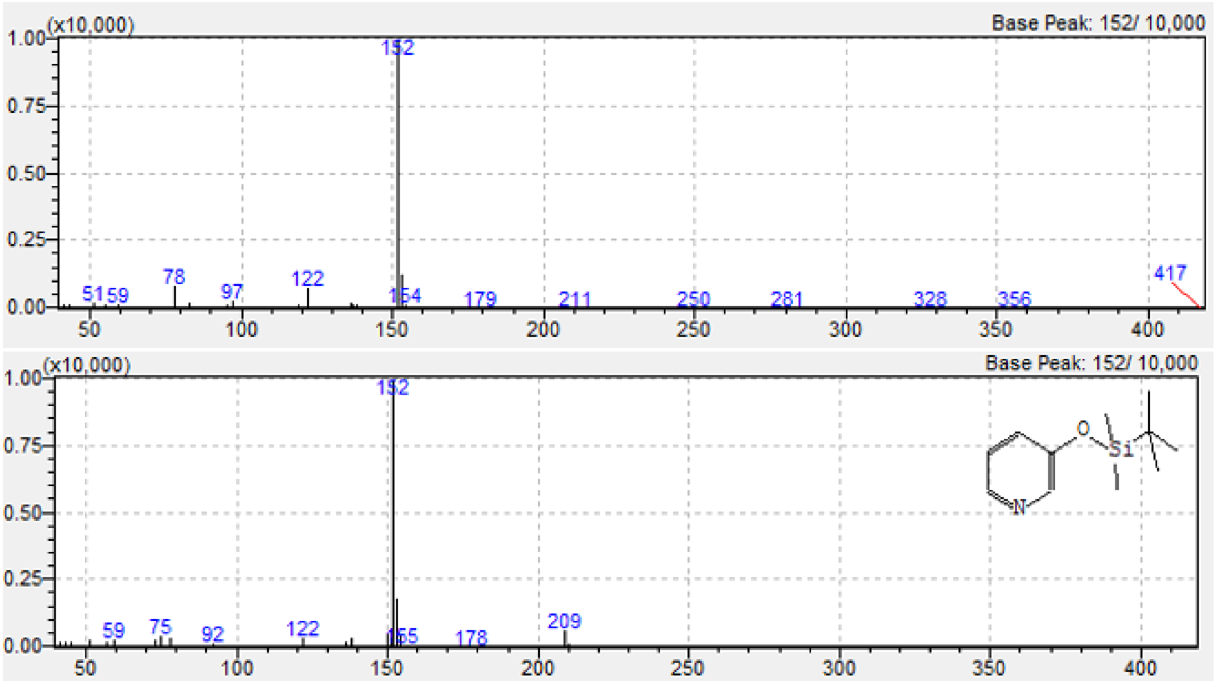

Figure S7. Mass-spectra of 3-tert-Butyldimethylsilyloxypyridine. First spectrum is of a typical run, second spectrum is from NIST.

Schemes

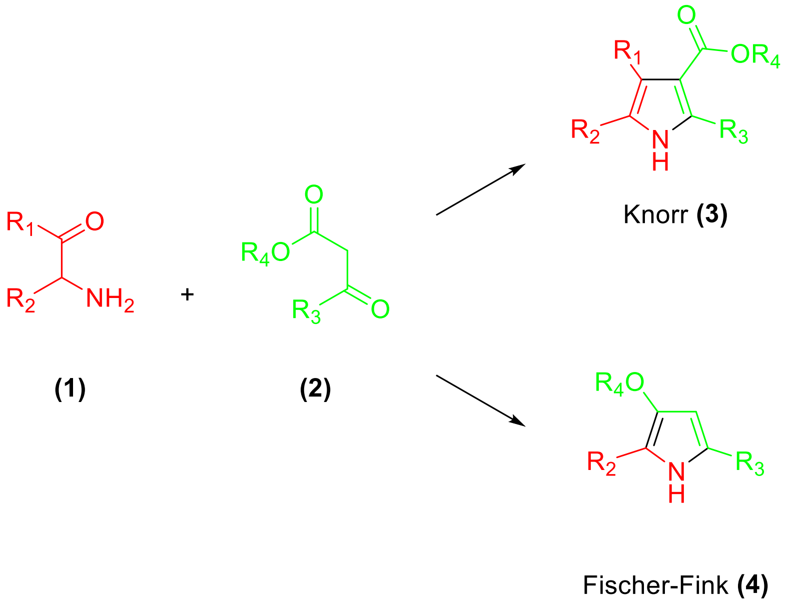

Scheme S1. Knorr-pyrrole synthesis. *Knorr*-product (4) as well as *Fischer-Fink*-product (5) can be observed in this reaction

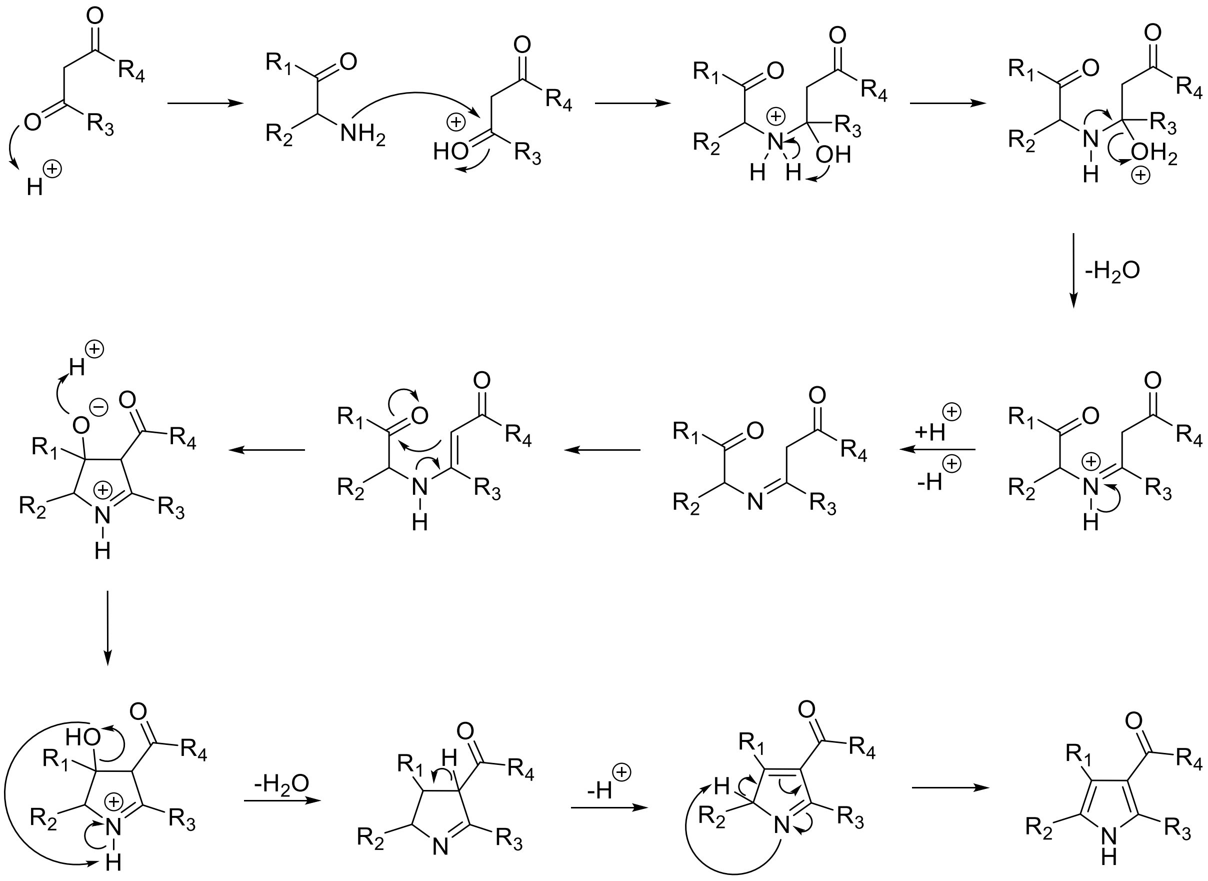

Scheme S2. Mechanism of the *Knorr*-pyrrole-synthesis

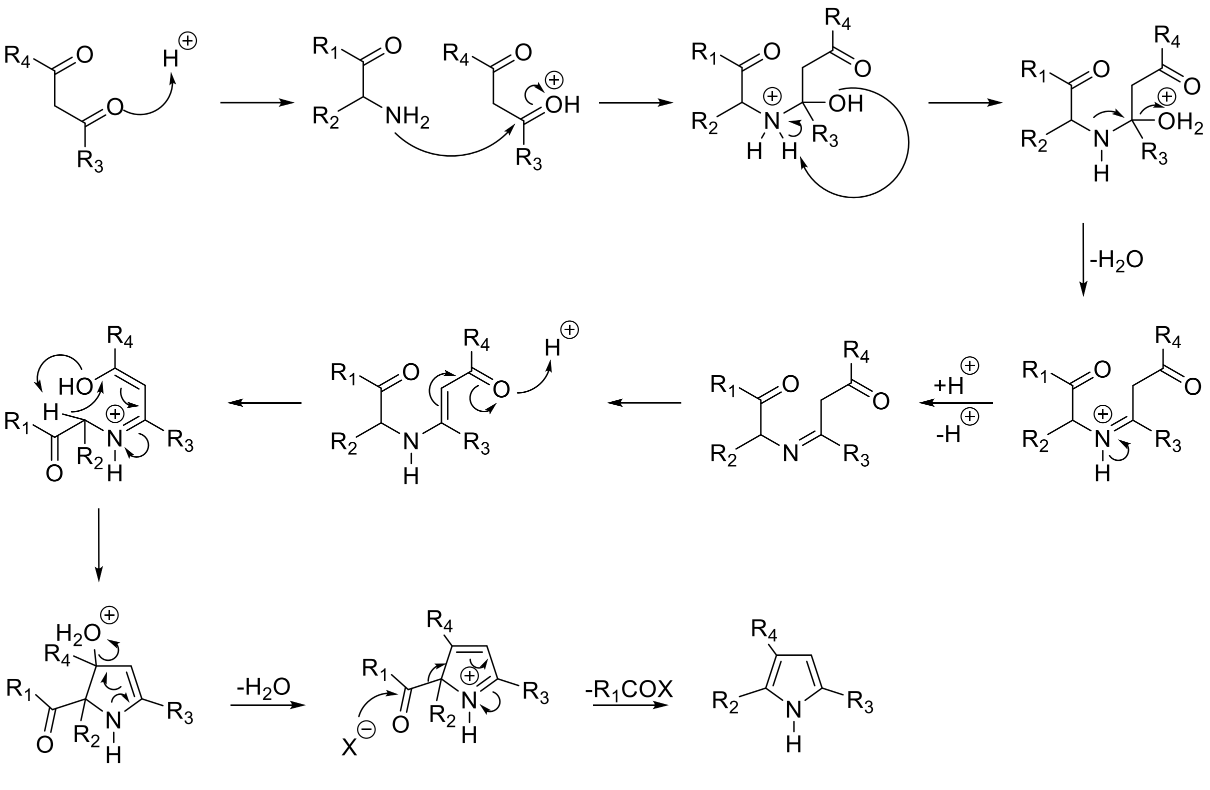

Scheme S3. Mechanism of the *Fischer-Fink*-pyrrole-synthesis

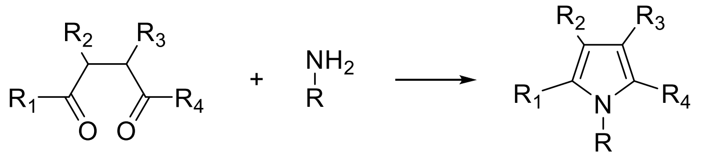

(5) **(6)** **(7)**

Scheme S4. *Paal-Knorr*-reaction.

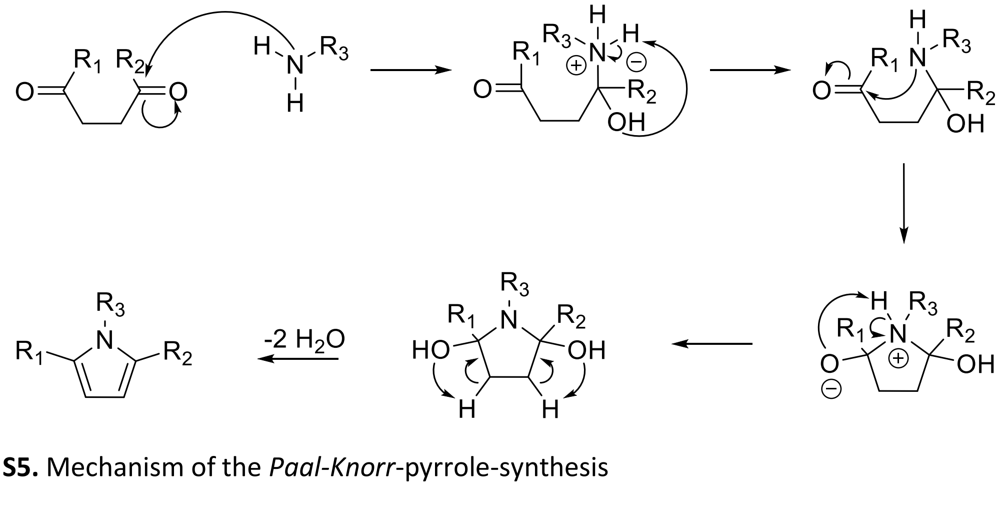

Scheme S5. Mechanism of the *Paal-Knorr*-pyrrole-synthesis

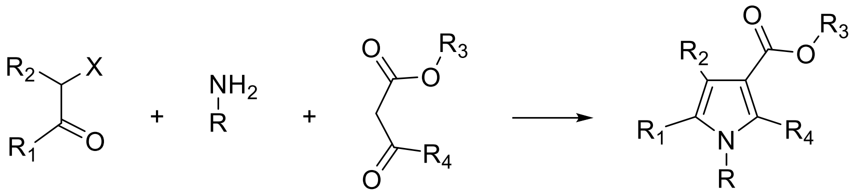

(8) **(6)** **(9)** **(10)**

Scheme S6. Pyrrole synthesis according to *Hantzsch*.

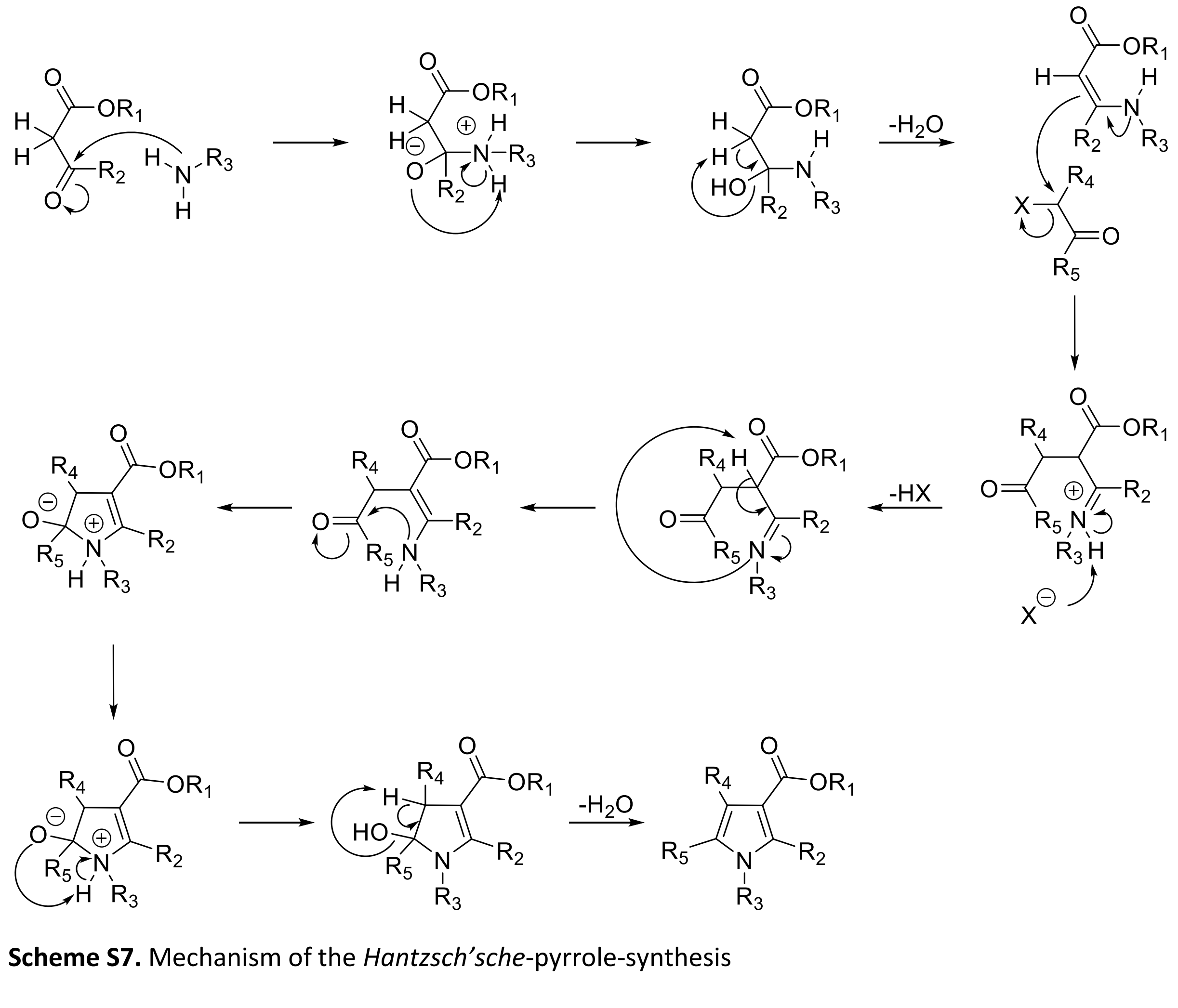

Scheme S7. Mechanism of the *Hantzsch'sche*-pyrrole-synthesis

Tables

If not stated otherwise, all runs were performed with 1.00 mmol $NiSO_4$, 1.00 mmol Na_2S, 1 mL NaOH, 60 mL CO and 60 mL acetylene for one day at 105 °C. A total reaction volume of 5 mL was achieved by adding argon-saturated water. Yields are given at mol% conversion based on NH_4Cl. pH was measured at the end of the reaction time.

Table S1. Formation of pyrrole depending on pH. Different values were achieved by adding different amounts of 1 M NaOH (run 5, 7, 8, 10, 11, 12, 14), $Ca(OH)_2$ (run 6, 9, 10), 1 M H_2SO_4 (run 1-3) or none of them (run 4). Other parameters as stated above. The defined standard run is run 8 in this table (1 mL NaOH (1M)).

Run	pH	NaOH (1 M)	$Ca(OH)_2$	H_2SO_4 (1 M)	pyrrole	yield
		[mmol]	[mmol]	[mmol]	[µM]	[%]
1	1.8	-	-	1	<0.1	<0.01
2	2.4	-	-	0.5	<0.1	<0.01
3	3.8	-	-	0.1	<0.1	<0.01
4	6.8	-	-	-	0.71	0.071
5	7.1	0.1	-	-	0.65	0.065
6	7.6	-	0.3	-	1.25	0.546
7	7.8	0.5	-	-	2.53	0.253
8	9.1	1.0	-	-	11.38	1.138
9	9.4	-	1.3	-	1.70	0.17
10	9.6	1.5	-	-	1.70	0.17
11	10.3	2.0	-	-	0.75	0.075
12	10.9	3.0	-	-	0.40	0.04
13	10.9	-	2.6	-	0.18	0.018
14	11.3	4.0	-	-	0.16	0.016

Table S2. Formation of pyrrole depending on reaction time. Other parameters as stated above. The defined standard run is run 19 in this table (1 d).

Run	t_{reac}	pH	pyrrole	yield
	[h]		[µM]	[%]
15	0.00	8.7	<0. 1	<0.01
16	0.08	8.8	<0. 1	<0.01
17	0.17	8.8	<0. 1	<0.01
18	0.50	8.7	<0. 1	<0.01
19	1.00	8.8	0.82	0.08
20	2.00	8.9	1.58	0.16
21	4.00	8.7	2.20	0.22
22	8.00	8.5	3.40	0.34

23	24.0	9.1	11.377	1.138
24	48.0	8.2	3.66	0.37
25	72.0	8.6	2.85	0.29
26	96.0	8.5	1.88	0.19
27	120	8.2	1.52	0.15
28	144	8.4	1.00	0.10
29	168	8.3	0.92	0.09

Table S3. Formation of pyrrole depending on concentration of catalyst. Different concentrations of NiS were achieved by adding different amounts of Na_2S to a constant amount of $NiSO_4$ (1 mmol). NiS, the actual catalyst is formed *in situ*. Other parameters as stated above. The defined standard run is run 34 in this table (1.00 mmol Na_2S).

Run	Na2S	pH	pyrrole	yield
	[mmol]		[µM]	[%]
30	0.00	6.9	<0.1	<0.01
31	0.25	7.2	<0.1	<0.01
32	0.50	7.5	0.76	0.08
33	0.75	7.9	0.62	0.06
34	1.00	9.1	11.377	1.138
35	1.25	9.0	4.78	0.48
36	1.50	9.4	2.63	0.26
37	1.75	9.8	0.57	0.06
38	2.00	11.1	0.32	0.03
